# Alterations of Cytokine Profiles in Patients With Recurrent Implantation Failure

**DOI:** 10.3389/fendo.2022.949123

**Published:** 2022-07-11

**Authors:** Ling Guo, Anliang Guo, Fang Yang, Li Li, Junhao Yan, Xiaohui Deng, Caifeng Dai, Yan Li

**Affiliations:** ^1^ Center for Reproductive Medicine, Shandong University, Jinan, China; ^2^ Key Laboratory of Reproductive Endocrinology of Ministry of Education, Shandong University, Jinan, China; ^3^ Medical Integration and Practice Center, Shandong University, Jinan, China; ^4^ Center for Reproductive Medicine, Department of Obstetrics and Gynecology, Qilu Hospital of Shandong University, Jinan, China; ^5^ Suzhou Research Institute, Shandong University, Suzhou, China

**Keywords:** *in vitro* fertilization, recurrent implantation failure, cytokines, Th1/Th2, logistic regression analysis

## Abstract

Serum cytokine profile and T helper (Th)1/Th2 cell balance are related to the success of embryo implantation, although not yet firmly linked to recurrent implantation failure (RIF), a repeated failure to achieve clinical pregnancy following multiple high-quality embryo transfer. In this prospective study, comprehensive bioinfomatic analysis and logistic regression analysis were used to compare the serum cytokine profiles of 41 RIF patients with those of 29 subjects with first-cycle successful pregnancy in the mid-luteal phase and to assess the alterations of cytokine profiles in patients with clinical pregnancy at five weeks post-transplantation. We found several elevated pro-inflammatory cytokines, decreased anti-inflammatory cytokines, and increased Th1/Th2 cytokine ratios in RIF patients compared to control subjects. Specifically, the receiver operating characteristic (ROC) curve generated using multiple indicators provides a high predictive value for diagnosing RIF (area under the curve [AUC] = 0.94, 95% confidence interval [CI] 0.87-1.00, *P* < 0.0001), with a sensitivity of 96.55% and a specificity of 87.50%. Meanwhile, at five weeks post-transplantation, patients in both groups diagnosed with clinical pregnancy exhibited increased levels of several cytokines compared with pre-pregnancy levels, and a gradual shift in Th1/Th2 balance toward Th2. These findings suggest that inflammatory serum cytokines and the predominance of Th1 cells likely contribute to RIF and possibly reflect the immune environment at the maternal-fetal interface, suggesting their value as outcome indicators in assisted reproductive therapy.

## Introduction

In China, the incidence of infertility has reached as high as 15% in recent years, becoming the third most common medical problem affecting human health after tumors and cardiovascular diseases (1, 2). To address fertility-related issues, assisted reproductive technology (ART), especially *in vitro* fertilization-embryo transfer (IVF-ET) and derivative technologies, has developed rapidly and has become the most widely used approach for the treatment of infertility patients. Currently, the cumulative live birth rate following IVF-ET is approximately 40% worldwide, and rates higher than 50% have been reported in some regions and reproductive centers (3). However, a large proportion of infertility patients still repeatedly fail to achieve a clinical pregnancy after multiple *in vitro* fertilization (IVF) cycles with high-quality embryos transferred. This outcome is termed recurrent implantation failure (RIF) and accounts for 5% to 10% of ART failures (4). Although the diagnostic criteria for RIF have not yet been unified, the currently accepted definition of RIF based on clinical research is “the failure to achieve a clinical pregnancy after transfer of at least four high-quality embryos in a minimum of three fresh or frozen cycles in a woman under the age of 40 years” (5). RIF thus poses one of the most urgent challenges facing the field of assisted reproduction, and it often results in severe physical and mental suffering for patients and their families. However, the pathogenesis of RIF remains unclear, and limited information is available regarding its indicators.

The etiology of RIF is complex and multifactorial, with contributions from maternal, embryonic, and external factors. Among them, maternal contributors include psychological factors, abnormalities in the reproductive system, aberrant endometrial development and function, and immunologic dysfunction at the maternal-fetal interface (6, 7). In particular, recent research efforts in reproductive immunology have focused on immune factors at the maternal-fetal interface, especially the role of peripheral blood immune cells in maternal-fetal communication and maternal remodeling. Despite these advances, the specific mechanisms governing this remodeling and its role in RIF remain unclear. Currently, the majority of studies on this topic have concluded that a variety of cytokines participate in the formation of maternal-fetal immune tolerance (8–10). Since the embryo acts as an allogeneic antigen, the cytokine profile in peripheral blood can significantly affect the stability of the immune system at the maternal-fetal interface during the implantation period, whether by direct or indirect regulatory roles. However, the expression patterns of the cytokine profile and its possible roles in the pathogenesis of RIF are poorly understood.

T lymphocytes, which account for approximately 60% of peripheral blood lymphocytes, originate from bone marrow lymphocyte stem cells, which differentiate and mature into immune cells capable of exerting immune activity in the thymus. Studies have found that the immune response mediated by T lymphocytes is an essential step in the process of embryo implantation (11, 12). In particular, CD4+ T and CD8+ T lymphocytes, defined by their characteristic expression of surface markers, are the predominant cell types found at the maternal-fetal interface. Among these cell types, helper T (Th) cells represent an independent subset of CD4+ T cells that have come to be recognized for their vital role in immune regulation at the maternal-fetal interface (13, 14). Th cells can be further categorized into Th1, Th2, Th17 and other subsets based on their expression of nuclear transcription factors, cytokines, and other functional characteristics (15). Notably, the dynamic balance of Th1/Th2 cells is a determining factor in immune system homeostasis at the maternal-fetal interface and consequently the pregnancy outcomes after IVF-ET, such as RIF (13, 14). Thus, this ratio could potentially serve as an effective indicator of maternal immune status during the implantation window period (10). However, relatively few studies have investigated the balance of Th1/Th2 cells in the peripheral blood of RIF patients, and as a result, the effects of these cells on IVF-ET remain controversial.

Therefore, to characterize the cytokine profiles of RIF patients, in this prospective study, we applied AimPlex multiplex immunoassays to detect serum levels of interleukin-2 (IL-2), IL-4, IL-6, IL-10, IL-17A, interferon-γ (IFN-γ), tumor necrosis factor-α (TNF-α), TNF-β, granulocyte colony-stimulating factor (G-CSF), and granulocyte-macrophage colony-stimulating factor (GM-CSF) in the middle luteal phase of 41 patients with RIF for comparison with those of 29 control subjects, with concurrent evaluation of differences in cytokines related to the ratio of Th1/Th2. We continued to follow up with the enrolled participants until five weeks after transplantation and compared the serum cytokine profiles of 10 patients with RIF and 14 controls who successfully achieved clinical pregnancy to identify pregnancy-associated changes in serum cytokine profiles. Thus, this study also aimed to identify cytokine-based biomarkers for RIF to facilitate the improvement of its clinical management through the development of more accurate diagnostics and targeted therapeutics.

## Materials and Methods

### Participants

In this prospective study, a total of 41 patients with RIF and 29 control subjects with first-cycle successful pregnancy were enrolled from patients undergoing IVF/intracytoplasmic sperm injection (ICSI) cycles at the Center for Reproductive Medicine of Qilu Hospital of Shandong University from March, 2020 to December, 2020. The inclusion criteria for RIF patients were as follows: failure to achieve a clinical pregnancy after transfer of at least four high-quality embryos or underwent a minimum of three fresh or frozen cycles, age < 40 years old, basal follicle-stimulating hormone (FSH) < 10 mIU/ml, and antral follicle counts (AFCs) in both ovaries ≥ 5. The criteria for inclusion of controls were the same as those for RIF cases except that the included patients had no history of adverse maternal-fetal outcomes and achieved clinical pregnancy after the first IVF/ICSI-ET cycle. The exclusion criteria for all patients were chronic autoimmune disease, abnormal anatomy of the genital tract, chromosomal anomalies in either the female or male prospective parents, endometrial thickness < 7 mm on the day of embryo transfer, incomplete clinical data and other factors that may affect the inflammatory process.

### IVF Procedure

All subjects included in this study adopted a standardized ovarian stimulation protocol, such as GnRH-agonist long-term protocol, ultra-long protocol, short-term protocol, micro-stimulation protocol, and GnRH-antagonist protocol. Gonadotropin (Gn) was used individually based on evaluations of age, body mass index (BMI), and ovarian function. Follicular development was monitored by transvaginal ultrasound. Human chorionic gonadotropin (hCG) was injected to induce ovulation when there were at least two dominant follicles with an average diameter of ≥18 mm. Oocyte retrieval was performed at 34-36 hours (h) after hCG administration. Fertilization by IVF or ICSI was selected depending on sperm quality at 4-6 h after oocyte retrieval. Cleavage-stage embryos were evaluated into four grades by morphological criteria based on the regularity of blastomeres and the degree of fragmentation (16). In our center, embryos (grades I-III) are considered as transferable embryos. All patients enrolled in this study underwent frozen-thawed embryo transfer (FET). All transferable embryos were further cultured until the blastocyst stage with sequential media. All blastocysts were scored by Gardner morphological criteria (17) and cryopreserved by vitrification on day 5 or day 6 for later transfer. In our center, the embryos of grades over 4BC according to Gardner morphological criteria were defined as high-quality embryos. At the second menstrual cycle after oocyte retrieval, endometrial preparation was performed either with a natural cycle regimen or a programmed cycle regimen. After the frozen embryos were recovered, up to two high-quality embryos were transferred in each cycle during the implantation period. Biochemical pregnancy was determined by serum β-hCG >10 IU/L on days 10 to 14 after embryo transfer. Clinical pregnancy was defined by transvaginal ultrasound detection of the intrauterine gestational sac at five weeks after embryo transfer.

### Sample Collection

In the mid-luteal phase prior to FET treatment, 5 ml fasting blood samples were collected from the anterior elbow vein of all subjects (including 41 RIF cases and 29 control cases). At five weeks after embryo transfer, blood samples were again drawn from patients diagnosed with clinical pregnancy. Among all subjects, as of the end of this study, 18 cases in the RIF group obtained clinical pregnancy, 10 of whom provided blood samples and complete follow-up data after pregnancy; blood samples were also obtained from 14 control cases after pregnancy. Blood samples were then centrifuged for 10 minutes at, 3000 X rpm. The serum was separated, and the supernatant was stored at −80°C for later analysis.

### Cytokine Profiling

Individual serum samples were subjected to cytokine profile quantification by AimPlex multiplex immunoassay kits (Beijing Quantobio, China). The kit protocol for quantitative analysis included 10 total cytokines, including IL-2, IL-4, IL-6, IL-10, IL-17A, IFN-γ, TNF-α, TNF-β, G-CSF, and GM-CSF. Briefly, 45 μL of capture bead working suspension was mixed with 45 μL of sample and shaken at 700 rpm for 60 min in the dark, followed by three washes with 100 μL 1 × wash buffer. Next, 25 μL of biotin-conjugated antibodies was added to each well, and the wells were sealed and shaken for 30 min in the dark. Then, after three washes with 1× wash buffer, 25 μL of streptavidin-PE was added to each well and shaken for 20 min in the dark. Finally, 100-200 μL of 1× reading buffer was added to each well, the fluorescence signals in each sample were acquired using a Navios flow cytometer (Beckman Coulter, USA), and the cytokine levels were analyzed with FCAP Array 3.0 software.

### Statistical Analysis

Statistical analysis was performed using SPSS 26.0 for Windows (IBM, Armonk, NY, USA) and GraphPad Prism 8.3 (GraphPad Software, San Diego, CA, USA). Principal component analysis (PCA), heatmap and cluster analysis, and forest map were conducted using the website www.bioinformatics.com.cn. Normality of the data distributions was assessed by Kolmogorov–Smirnov tests. Data are presented as the means ± standard deviations (for normally distributed data) or medians with interquartile range (for nonnormally distributed data). Continuous variables with normal distributions were analyzed by independent *t test* and data with a non-normal distribution were compared by the Mann–Whitney U test. For paired comparisons, Wilcoxon paired test was used to compare the significant differences. Categorical variables are presented as counts (percentages) and were compared using either the chi-square or Fisher’s exact test. Selected variables were entered into multivariable logistic regression analysis to assess predicting factors for RIF and compute odds ratios (ORs) with 95% confidence intervals (CIs) for each endpoint. Specificity and sensitivity were assessed using receiver operating characteristic (ROC) analysis. In all analyses, *P* < 0.05 was considered statistically significant.

## Results

### Baseline Characteristics and Related Clinical Indicators

In total, 41 patients with RIF and 29 control patients who met the inclusion criteria were enrolled. Among the 41 patients in the RIF group, the average number of transfer failures was 3.12 ± 1.25, and the cumulative number of embryos transferred was 3.90 ± 1.87. As shown in **Table 1**, there were no significant differences between the RIF and control groups in the type of infertility, BMI, anti-Mullerian hormone (AMH), basal FSH, basal luteinizing hormone (LH), basal estradiol (E2), basal progesterone (P), basal prolactin (PRL), basal testosterone (T), free triiodothyronine (FT3), free thyroxin (FT4), thyroid-stimulating hormone (TSH), thyroglobulin antibody (TG-Ab), or thyroid peroxidase antibody (TPO-Ab) (*P* > 0.05); however, age was significantly higher in the RIF group, while endometrial thickness on the day of embryo transfer was lower in RIF patients than in the control group, which is in agreement with clinical assessments.

**Table 1:** Baseline characteristics and related clinical indicators of the participants.

Characteristics	RIF group (*N* = 41)	Control group (*N* = 29)	*P* values
No. of transfer failure	3.12 ± 1.25		
No. of embryo transferred	3.90 ± 1.87		
Age (years)	32.54 ± 3.81	29.07 ± 4.05	0.001*
Type of infertility (n, %)			0.225
Primary	18 (43.90%)	17 (58.62%)	
Secondary	23 (56.10%)	12 (41.38%)	
BMI (kg/m^2^)	22.28 ± 2.41	22.12 ± 2.89	0.802
AMH (ng/ml)	3.36 (2.31-4.90)	3.73 (2.65-5.25)	0.183
Basal FSH (IU/L)	6.65 (5.61-7.74)	6.32 (5.41-7.89)	0.642
Basal E2 (pg/ml)	35.83 (26.65-47.16)	39.72 (32.83-51.62)	0.081
Basal P (ng/ml)	0.28 (0.17-0.37)	0.21 (0.10-0.38)	0.387
Basal PRL (ng/ml)	17.24 (14.17-23.24)	18.35 (15.21-24.04)	0.486
Basal LH (IU/L)	4.68 (3.62-6.66)	6.34 (4.60-7.20)	0.060
Basal T (ng/ml)	0.23 (0.20-0.30)	0.28 (0.21-0.43)	0.085
FT3 (pmol/L)	4.80 ± 0.62	4.67 ± 0.47	0.345
FT4 (pmol/L)	16.78 ± 2.95	17.11 ± 1.61	0.580
TSH (μIU/mL)	2.49 ± 1.05	2.59 ± 1.23	0.711
TG-Ab (IU/ml)	10.00 (10.00-10.00)	10.00 (10.00-13.41)	0.329
TPO-Ab (IU/ml)	7.99 (5.00-11.37)	9.71 (7.42-13.53)	0.056
Endometrial thickness (mm)	9.32 ± 1.67	10.32 ± 1.90	0.024*

Data are presented as the mean ± standard deviation, median (interquartile range) or n (%). P < 0.05 was considered statistically significant and indicated by an asterisk. BMI, body mass index; AMH, anti-Mullerian hormone; FSH, follicle-stimulating hormone; E2, estradiol; P, progestogen; PRL, prolactin; LH, luteinizing hormone; T, testosterone; FT3, free triiodothyronine; FT4, free thyroxin; TSH, thyroid-stimulating hormone; TG-Ab, thyroglobulin antibody; TPO-Ab, thyroid peroxidase antibody.

### Controlled Ovarian Hyperstimulation and Laboratory Indicators

The numbers of participants in each group given different controlled ovarian hyperstimulation (COH) and fertilization procedures are compared in **Table 2**. There were no significant differences between the RIF and control groups in fertilization method, Gn duration, number of oocytes retrieved, number of metaphase II (MII) oocytes, number of normal fertilized oocytes, number of transferable embryos or number of high-quality embryos (*P* > 0.05).

**Table 2:** Controlled ovarian hyperstimulation and laboratory indicators.

Characteristics	RIF group (*N* = 41)	Control group (*N* = 29)	*P* values
Fertilization (n, %)			0.588
IVF	32 (78.05)	21 (72.41)	
ICSI	9 (21.95)	8 (27.59)	
Gn duration (days)	9.00 (7.00-10.00)	8.00 (7.00-9.00)	0.127
No. of oocytes retrieved	12.23 ± 6.21	13.86 ± 7.26	0.323
No. of MII oocytes	11.49 ± 5.96	11.10 ± 5.26	0.784
No. of normal fertilized oocytes	8.36 ± 4.44	9.41 ± 4.21	0.326
No. of transferable embryos	5.00 ± 2.59	5.69 ± 3.08	0.320
No. of high-quality embryo	3.74 ± 2.45	4.72 ± 3.00	0.143

Data are presented as the mean ± standard deviation, median (interquartile range) or n (%). P < 0.05 was considered statistically significant. IVF, in vitro fertilization; ICSI, intracytoplasmic sperm injection; Gn, gonadotropin; MII, metaphase II.

### Serum Cytokine Profiles

**Figure 1:**
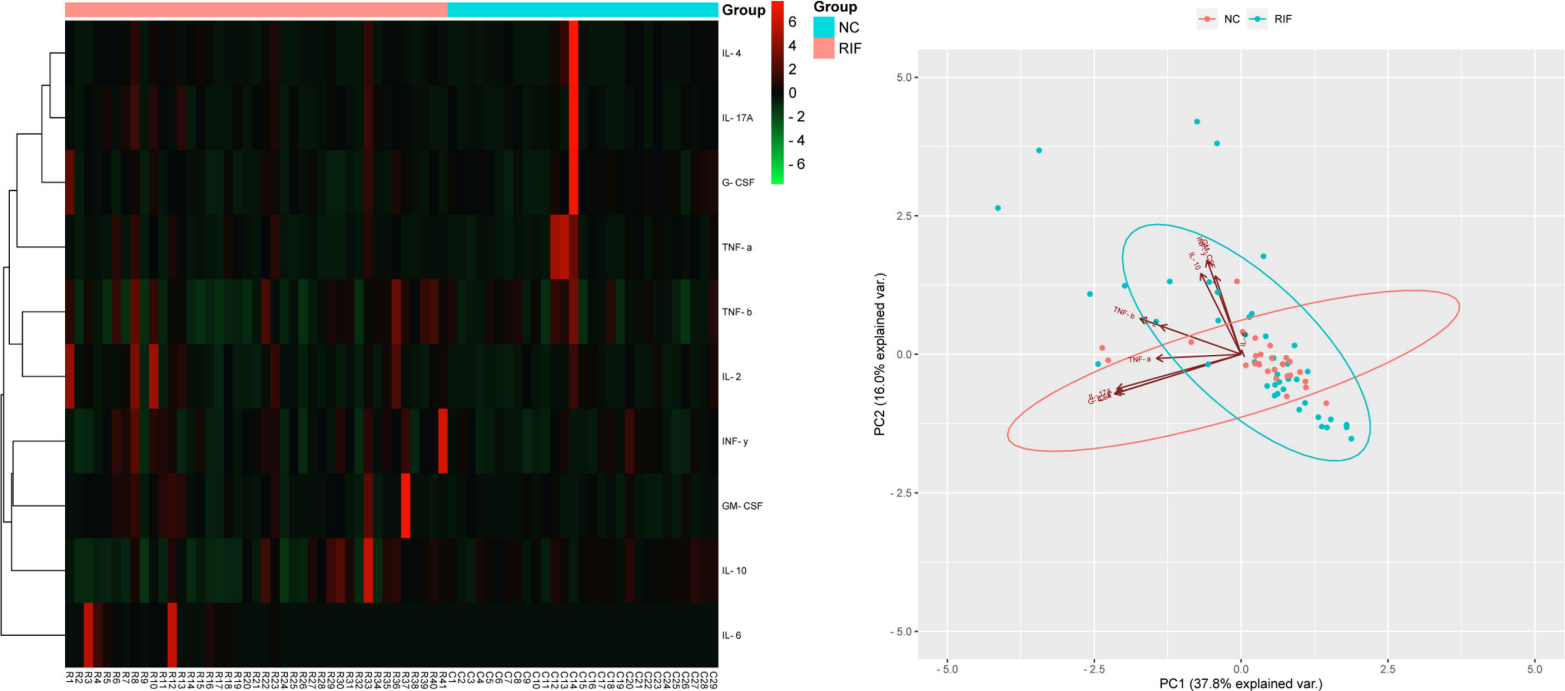
Cytokine profile screening with heatmap and PCA. Heatmap and PCA of 10 cytokine profiles in serum. Each row in the heatmap represents an individual differentially expressed cytokine, and the column displays the sample number. Cytokines with lower levels are displayed in green, while cytokines with higher levels are displayed in red. The points in the PCA diagram represent the samples, red for the control group and blue for the RIF group. The arrows represent the contribution and correlation of the corresponding original variables to the principal component.

**Figure 2:**
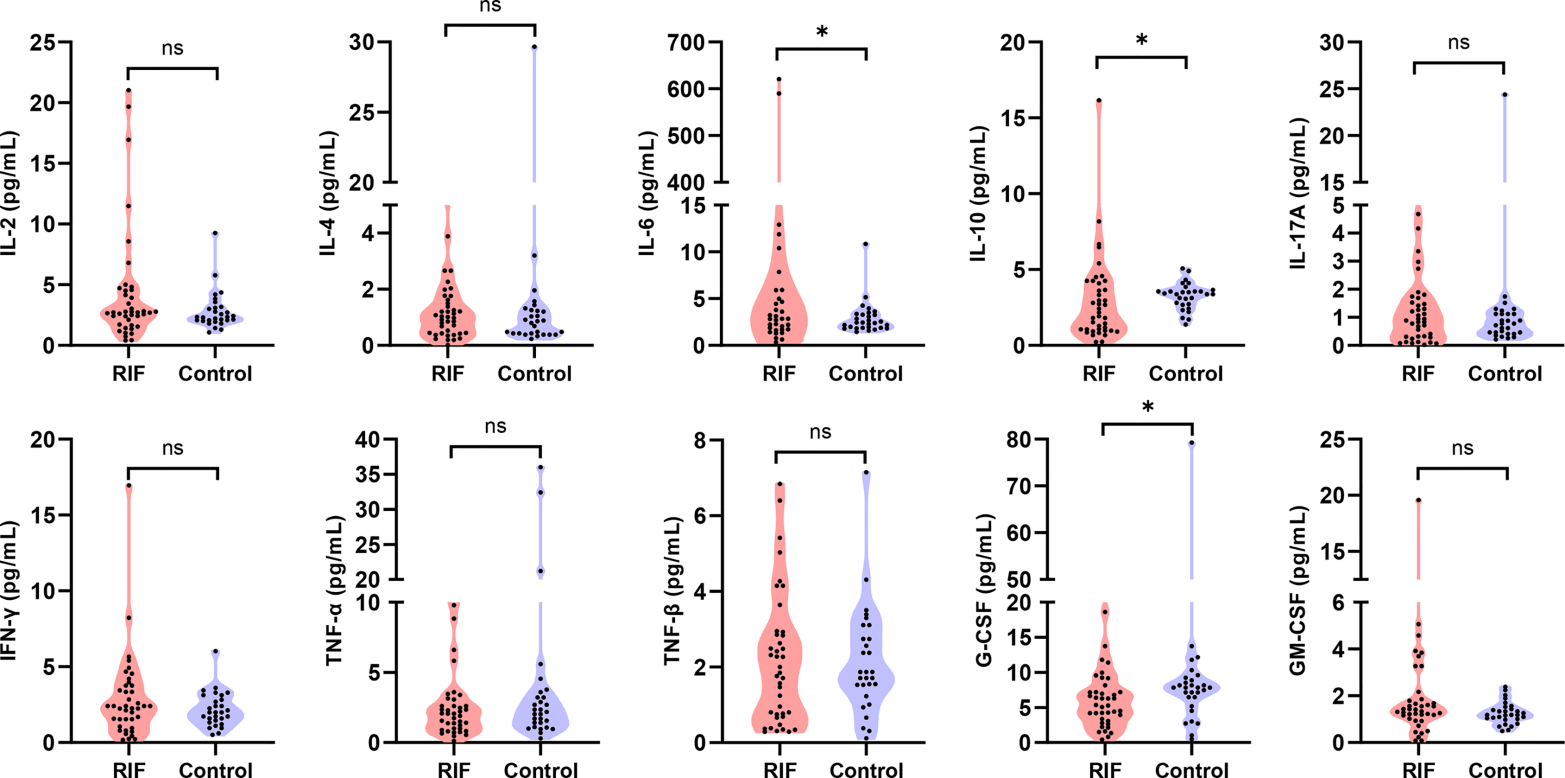
Serum cytokine profiles in the mid-luteal phase between RIF patients and control women. Every different cytokine is shown as density (violin shap), and data are shown as the median (interquartile range) for the two groups. *P < 0.05; **P < 0.01; ***P < 0.001; ****P < 0.0001; NS, not significant.

In the mid-luteal phase prior to FET treatment, a panel of 10 total cytokines were measured for comparison of their levels between 41 women with RIF and 29 controls. The results indicated that several serum markers accumulated to different levels between the two groups and that a variety of cytokines contributed to first principal component (PC1), which may serve as the main reason for the variation (**Figure 1**). In particular, the levels of IL-6 were significantly higher in the RIF group, while IL-10 and G-CSF were both lower than those in the control group (*P* < 0.05). In addition, IL-2, IL-4, IL-17A, IFN-γ, and GM-CSF levels were all higherin the peripheral blood samples of the RIF group, whereas TNF-α and TNF-β showed lower, but nonsignificant (*P* > 0.05), compared with control group levels (**Table 3**, **Figure 2**).

**Table 3:** Serum cytokine profiles in the mid-luteal phase.

Cytokines (pg/mL)	RIF group (*N* = 41)	Control group (*N* = 29)	*P* values
IL-2	2.71 (1.69-4.55)	2.35 (2.04-3.14)	0.319
IL-4	1.01 (0.44-1.70)	0.86 (0.43-1.29)	0.604
IL-6	3.61 (1.97-16.48)	2.45 (1.88-3.33)	0.042*
IL-10	2.18 (1.02-4.17)	3.37 (2.70-3.61)	0.034*
IL-17A	0.94 (0.32-1.72)	0.78 (0.44-1.12)	0.445
IFN-γ	2.40 (1.30-3.76)	1.99 (1.40-2.98)	0.407
TNF-α	2.06 (1.00-3.11)	2.19 (1.26-3.41)	0.428
TNF-β	1.85 (0.72-2.90)	1.87 (1.38-2.93)	0.698
G-CSF	5.36 (3.24-7.69)	7.83 (5.83-9.06)	0.033*
GM-CSF	1.41 (1.09-2.01)	1.18 (1.00-1.48)	0.111

Data are presented as the median (interquartile range). P < 0.05 was considered statistically significant and indicated by an asterisk. IL, interleukin; IFN, interferon; TNF, tumor necrosis factor; G-CSF, granulocyte colony-stimulating factor; GM-CSF, granulocyte-macrophage colony-stimulating factor.

To better understand how changes in cytokine levels before and after pregnancy may be related to the occurrence of RIF, we also collected peripheral blood samples from 10 patients in the RIF group and 14 patients in the control group at five weeks after embryo transfer who successfully achieved clinical pregnancy. The results showed that these patients in both the RIF and control groups displayed an overall increasing trend in cytokine levels from pre- to during-pregnancy. Among them, IL-10 and TNF-α levels in peripheral blood were significantly higher after clinical pregnancy than before pregnancy in the RIF group (*P* < 0.05) (**Figure S1A**). Meanwhile, in the control group, IL-6 concentrations were higher in peripheral blood samples after clinical pregnancy than that before pregnancy (*P* < 0.05) (**Figure S1B**).

### Serum Th1/Th2 Ratios

This study also investigated differences in the Th1/Th2 ratio between the two groups by determining the ratio of Th1-/Th2-associated cytokines in the peripheral blood of patients. In particular, IL-2, IFN-γ, TNF-α and TNF-β are pro-inflammatory cytokines secreted by Th1 cells, while IL-4 and IL-10 are anti-inflammatory cytokines specifically secreted by Th2 cells. The results showed that the IL-2/IL-10 and IFN-γ/IL-10 ratios in the RIF group were higher than those in the control group (*P* < 0.05), which suggested a relationship between these proportions and the occurrence of RIF. However, we found no significant differences in other ratios, although the RIF group showed a trend of higher Th1/Th2 cytokine ratios than the control group (**Table 4**).

**Table 4:** Serum Th1/Th2 ratios in the mid-luteal phase.

Characteristics	RIF group (N = 41)	Control group (N = 29)	*P* values
IL-2/IL-4	3.03 (1.89-5.71)	2.95 (2.02-5.54)	0.986
IL-2/IL-10	1.35 (0.69-2.57)	0.74 (0.60-1.07)	0.007*
IFN-γ/IL-4	2.00 (1.15-4.41)	2.19 (1.47-4.50)	0.825
IFN-γ/IL-10	0.95 (0.56-1.92)	0.58 (0.44-0.96)	0.012*
TNF-α/IL-4	2.03 (1.35-3.84)	2.94 (2.04-4.92)	0.075
TNF-α/IL-10	1.14 (0.53-1.47)	0.70 (0.51-0.97)	0.180
TNF-β/IL-4	1.78 (0.90-4.23)	1.74 (1.14-3.97)	0.761
TNF-β/IL-10	0.76 (0.48-1.18)	0.61 (0.36-0.88)	0.147

Data are presented as the median (interquartile range). P < 0.05 was considered statistically significant and indicated by an asterisk. IL, interleukin; IFN, interferon; TNF, tumor necrosis factor.

The cytokines were also quantified to calculate the Th1/Th2 ratios in pre- and during-pregnancy samples. The results showed that these patients in both the RIF and control groups displayed an overall decreasing trend in the Th1/Th2 ratio from pre- to during-pregnancy. Among them, the IL-2/IL-10 ratio was significantly lower after clinical pregnancy than before pregnancy in the RIF group (*P* < 0.05) (**Figure S1C**). Meanwhile, in the control group, the TNF-α/IL-4 ratio was significantly lower after clinical pregnancy than before pregnancy (*P* < 0.05) (**Figure S1D**).

### Logistic Regression Analysis and ROC Curve Evaluation

**Figure 3:**
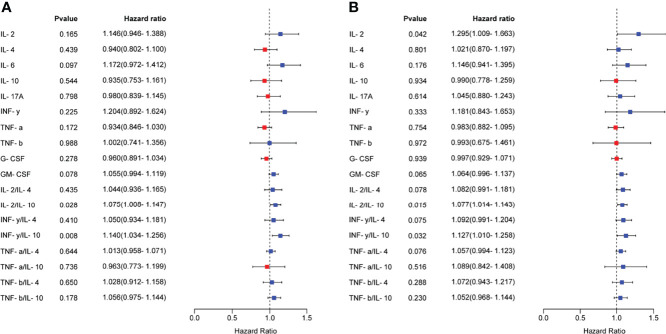
Relationships between serum cytokine profiles and the occurrence of RIF. **(A)**, The relationship between the serum cytokine profiles in the mid-luteal phase and the occurrence of RIF was analyzed by univariate logistic regression analysis and visualized as a forest map. **(B)**, After correcting for maternal age and endometrial thickness on the day of embryo transfer, the relationship between the serum cytokine profiles and the occurrence of RIF was analyzed by logistic regression analysis. OR > 1 indicates a risk factor, and OR < 1 indicates a protective factor.

**Figure 4:**
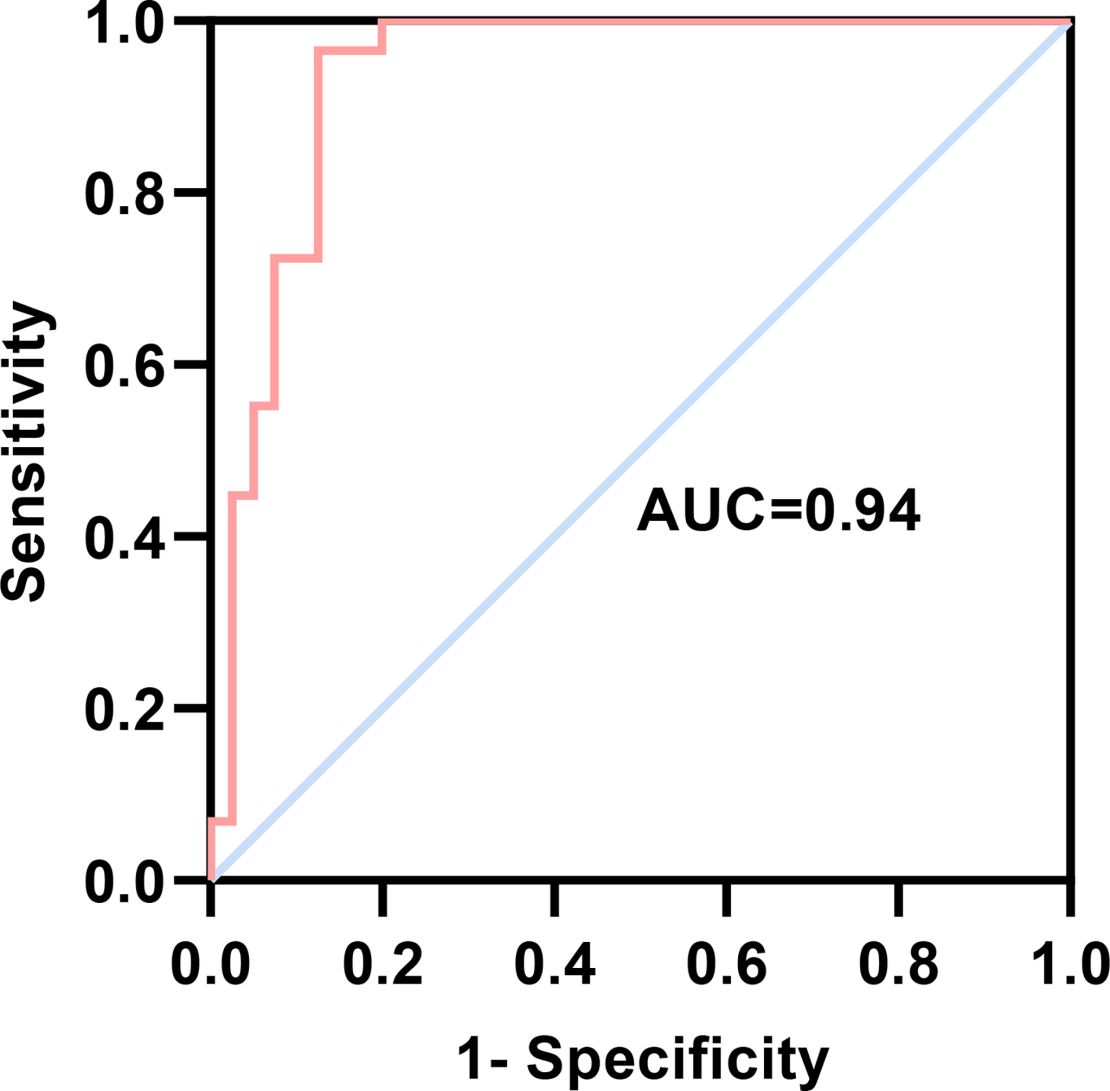
ROC curve evaluation of the logistic regression model. The AUC of the predictive model was 0.94 (95% CI 0.87-1.00, *P* < 0.0001), with a sensitivity of 96.55% and a specificity of 87.50%. AUC, area under the curve; CI, confidence interval.

Logistic regression analysis was used to further explore the relationships between the serum cytokine profiles during the mid-luteal phase and the occurrence of RIF. We found that several cytokines and Th1/Th2 ratios were associated with RIF (**Figure 3A**). After adjusting for the influence of maternal age and endometrial thickness on the day of embryo transfer, IL-2, IL-2/IL-10 and IFN-γ/IL-10 were still found to have a high risk of developing RIF (**Figure 3B**). To generate a predictive model for RIF, we conducted multivariable logistic regression analysis of the associated indicators. We found that age, IL-10, G-CSF and IL-2/IL-10, in particular, had independent predictive values for the occurrence of RIF (*P* < 0.05) (**Table 5**). Then, the prediction probability based on the binary logistic regression model was determined by ROC curve analysis. Notably, the predictive model constructed using age, endometrial thickness, IL-6, IL-10, G-CSF, IL-2/IL-10 and IFN-γ/IL-10 had a good diagnostic performance (AUC = 0.94, 95% CI 0.87-1.00, *P* < 0.0001), with a sensitivity of 96.55% and a specificity of 87.50% (**Figure 4**).

**Table 5:** Logistic regression analysis of RIF risk prediction.

Characteristics	β	S.E.	Wald	*P* values	OR	95% CI
Age (years)	0.26	0.12	4.67	0.031*	1.30	(1.02,1.64)
Endometrial thickness (mm)	-0.24	0.22	1.14	0.286	0.79	(0.51,1.22)
IL-6 (pg/mL)	0.10	0.14	0.49	0.486	1.10	(0.84,1.44)
IL-10 (pg/mL)	0.67	0.24	7.69	0.006*	1.96	(1.22,3.14)
G-CSF (pg/mL)	-0.37	0.14	6.76	0.009*	0.69	(0.52,0.91)
IL-2/IL-10	2.25	0.91	6.06	0.014*	9.49	(1.58,56.97)
IFN-γ/IL-10	1.09	0.83	1.73	0.188	2.97	(0.59,15.05)
Constant	-9.19	4.52	4.13	0.042	0.00	

P < 0.05 was considered statistically significant and indicated by an asterisk. β, regression coefficient; S.E., standard error; OR, odds ratio; CI, confidence interval; IL, interleukin; G-CSF, granulocyte colony-stimulating factor; IFN, interferon.

## Discussion

Successful embryo implantation requires synchronized and coordinated interactions between the embryo and the maternal endometrium (18, 19). Previous studies have proposed that the immune cells in the endometrium and peripheral blood and their various associated cytokines constitute a unique cytokine immunoregulatory network, especially during the implantation window (20) (which occurs in the mid-luteal phase). During this period, the endometrial morphology and transcriptional profile of cytokines change. This event leads to immune cell aggregation and subsequent secretion of a diverse array of cytokines to regulate embryo positioning and implantation (21, 22). Thus, the formation of immune tolerance at the maternal-fetal interface is essential for achieving normal pregnancy, while an imbalance in the immune microenvironment will lead to failure of embryo implantation or miscarriage (23, 24).

However, the precise regulatory mechanism controlling cytokine profiles and the immune response at the maternal-fetal interface in RIF patients remains unknown. In the current study, we characterized the cytokine profiles of RIF patients using IVF/ICSI-ET cases that were successful in the first cycle as a control group. Our results show that various pro-inflammatory cytokines are elevated, whereas anti-inflammatory cytokines are decreased, and Th1/Th2 ratios are increased during the mid-luteal phase in RIF patients compared with the control group, suggesting that alterations in the serum cytokine profile during the middle luteal phase in particular may play a role in the occurrence of RIF. Specifically, ROC curves generated using several indicators established here provide high predictive value for diagnosing RIF. Moreover, several cytokines were increased in both groups after pregnancy, but the Th1/Th2 balance gradually shifted toward Th2 cells, which may contribute to embryo implantation and the maintenance of normal pregnancy.

Studies have reported that the participation of cytokines in the immune response at the maternal-fetal interface can significantly impact pregnancy outcomes after IVF-ET (25). Among these cytokines, IL-2, IFN-γ, TNF-α, and TNF-β are pro-inflammatory cytokines mainly secreted by Th1 cells. In particular, IL-2 and IFN-γ were found to synergistically activate the cytotoxic effects of T lymphocytes and NK cells, which can destroy trophoblasts and kill embryos (26). Interestingly, TNF-α could induce positive regulatory effects on trophoblast invasion at low concentrations (27). In contrast, high TNF-α concentrations can inhibit endometrial decidualization and promote apoptosis in trophoblasts, leading to embryo implantation failure or pregnancy loss (28, 29). Distinct from Th1 cytokines, IL-4, IL-6 and IL-10 cytokines are primarily secreted by Th2 cells. Among them, IL-4 and IL-10 can cooperate with Treg cells in the regulation of immune balance to ensure a conducive environment for embryo implantation and maintenance of pregnancy (30). Although IL-6 is secreted by Th2 cells, it belongs to the pro-inflammatory cytokine group and performs several diverse biological functions (31). Previous work has found that IL-6, at physiological concentrations, can promote trophoblast proliferation by activating matrix metalloproteinase (MMP), which facilitates embryo implantation. However, abnormally high expression of IL-6 is often associated with recurrent spontaneous abortion (RSA) and RIF (32–34). IL-17A is a pro-inflammatory cytokine secreted explicitly by Th17 cells, which can induce a robust inflammatory response and contributes to a variety of autoimmune diseases (35–37). G-CSF and GM-CSF are members of the colony-stimulating factor cytokine family, mainly secreted by activated monocytes and macrophages. These cytokines can positively regulate endometrial receptivity by promoting endometrial angiogenesis, which is crucial for embryo implantation (38, 39).

The results of our current study showed that IL-6 levels were significantly higher in the mid-luteal phase in RIF patients than in the control group, while IL-10 and G-CSF levels were significantly lower. Based on these findings, we tentatively conclude that aberrantly high serum IL-6 or insufficient levels of IL-10 and G-CSF during the peri-implantation period can adversely affect embryo implantation, suggesting that dysregulation of these cytokines is an immunological factor potentially leading to RIF. In addition, serum levels of IL-10 and TNF-α were significantly higher after pregnancy in the RIF group than before pregnancy, while during-pregnancy IL-6 levels were significantly higher than pre-pregnancy levels in the control group. It should be noted that we observed an overall increasing trend in the cytokine levels of both groups after pregnancy, which may suggest that a certain degree of inflammatory stimulation is required for successful embryo implantation.

Considerable research efforts have been dedicated to determining the role of the Th1/Th2 balance in maternal-fetal immune regulation. Previous reports have implicated Th1/Th2 imbalance in a variety of immune diseases, such as RSA (25), allergic bronchial asthma (40), and diabetes (41). However, the limited sample size, experimental design flaws, and inadequate follow-up of clinical trials have resulted in gaps in our understanding and persistent controversy surrounding the relationship between cytokine profiles and Th1/Th2 balance in the peripheral blood of RIF patients. Previous studies have concluded that the balance of Th1/Th2 shifts to Th2 cells during embryo implantation and pregnancy, resulting in a predominant Th2-type immune response, while the proportion of Th1 cells accordingly decreases (42). However, recent studies on this topic described a dynamic equilibrium in Th1/Th2 proportions. For example, a specific proportion of Th1 cells is necessary during embryo implantation to activate a slight inflammatory reaction at the maternal-fetal interface that is conducive to trophoblast invasion, tissue remodeling, and angiogenesis (25). The gradual shift toward predominant Th2 cell populations after embryo implantation likely inhibits the Th1-mediated immune response, thereby inducing immune tolerance that is beneficial to the maintenance of pregnancy (43).

Kwak-Kim (44) and Liang (45) found that Th1/Th2 cytokine ratios, such as IFN-γ/IL-4, IFN-γ/IL-10, TNF-α/IL-4, and TNF-α/IL-10, were significantly higher in the peripheral blood of RIF patients than in normal control subjects, suggesting that the immune response mediated by Th1 cells in peripheral blood may lead to RIF. Similarly, our results showed that the IL-2/IL-10 and IFN-γ/IL-10 cytokine ratios were markedly higher in the mid-luteal phase in the RIF group than in the control group and that after pregnancy, the IL-2/IL-10 ratio was lower in RIF patients, while TNF-α/IL-4 was lower in the control group than in pre-pregnancy values. We also noted an overall decreasing trend in Th1/Th2 cytokine ratios after pregnancy among patients in both groups. These collective findings strongly suggest that an obvious Th1/Th2 immune imbalance in the peripheral blood of IVF/ICSI-ET patients, particularly during the mid-luteal phase, could lead to embryo implantation failure, while an increasing Th1/Th2 bias toward Th2 cells may be beneficial to implantation and normal pregnancy.

Although there are many studies on cytokines or the Th1/Th2 balance at the maternal-fetal interface, most studies have only described the different expression signatures. In our study, the predictive model constructed using multiple indicators showed good diagnostic performance for RIF. However, due to the particularity of RIF, this study is limited by a relatively small cohort size, and further analysis with a larger sample size is needed to validate our conclusions.

In conclusion, this study reveals alterations in the serum cytokine profiles of RIF patients, suggesting that dysregulation of these cytokines during the middle luteal phase in particular is an essential factor potentially leading to RIF. Moreover, we identify a cytokine-based predictive model for RIF that is informative in the prediction, diagnosis and treatment of this common disease that accounts for ART failures.

## Data Availability Statement

The raw data supporting the conclusions of this article will be made available by the authors, without undue reservation.

## Ethics Statement

The studies involving human participants were reviewed and approved by the Medical Ethics Committee of Qilu Hospital of Shandong University. The patients/participants provided their written informed consent to participate in this study.

## Author Contributions

YL and CD conceived and designed the project. LG and AG performed the experiments and analyzed the data. FY, LL, JY and XD collected the clinical serum samples and information. LG and AG wrote the manuscript. JY, XD, CD, and YL critically revised the manuscript. All authors have been involved in interpreting the data and approved the final version.

## Funding

This study was supported by grants from the National Natural Science Foundation of China (82101784, 82171648), the Natural Science Foundation of Shandong Province (ZR2020QH051), the Natural Science Foundation of Jiangsu Province (BK20200223), and the Young Scholars Program and Fundamental Research Funds of Shandong University.

## Conflict of Interest

The authors declare that the research was conducted in the absence of any commercial or financial relationships that could be construed as a potential conflict of interest.

## Publisher’s Note

All claims expressed in this article are solely those of the authors and do not necessarily represent those of their affiliated organizations, or those of the publisher, the editors and the reviewers. Any product that may be evaluated in this article, or claim that may be made by its manufacturer, is not guaranteed or endorsed by the publisher.

## References

[1] Gurunath S Pandian Z Anderson RA Bhattacharya S. Defining Infertility--a Systematic Review of Prevalence Studies. Hum Reprod Update (2011) 17:575–88. doi: 10.1093/humupd/dmr015 21493634

[2] Montoya JM Bernal A Borrero C. Diagnostics in Assisted Human Reproduction. Reprod Biomed Online (2002) 5:198–210. doi: 10.1016/s1472-6483(10)61624-0 12419046

[3] Polanski LT Baumgarten MN Quenby S Brosens J Campbell BK Raine-Fenning NJ. What Exactly Do We Mean by ‘Recurrent Implantation Failure’? A systematic review and opinion. Reprod Biomed Online (2014) 28:409–23. doi: 10.1016/j.rbmo.2013.12.006 24581986

[4] Mak JSM Chung CHS Chung JPW Kong GWS Saravelos SH Cheung LP. The Effect of Endometrial Scratch on Natural-Cycle Cryopreserved Embryo Transfer Outcomes: A Randomized Controlled Study. Reprod Biomed Online (2017) 35:28–36. doi: 10.1016/j.rbmo.2017.04.004 28476486

[5] Coughlan C Ledger W Wang Q Liu F Demirol A Gurgan T. Recurrent Implantation Failure: Definition and Management. Reprod Biomed Online (2014) 28:14–38. doi: 10.1016/j.rbmo.2013.08.011 24269084

[6] Bashiri A Halper KI Orvieto R. Recurrent Implantation Failure-Update Overview on Etiology, Diagnosis, Treatment and Future Directions. Reprod Biol Endocrinol (2018) 16:121. doi: 10.1186/s12958-018-0414-2 30518389 PMC6282265

[7] Cakiroglu Y Tiras B. Determining Diagnostic Criteria and Cause of Recurrent Implantation Failure. Curr Opin Obstet Gynecol (2020) 32:198–204. doi: 10.1097/GCO.0000000000000620 32251092

[8] Chaichian S Shoaee S Saremi A Pedar S Firouzi F. Factors Influencing Success Rate of Leukocyte Immunization and Anti-Paternal Antibodies in Spontaneous Recurrent Miscarriage. Am J Reprod Immunol (2007) 57:169–76. doi: 10.1111/j.1600-0897.2006.00451.x 17295895

[9] Tisoncik JR Korth MJ Simmons CP Farrar J Martin TR Katze MG. Into the Eye of the Cytokine Storm. Microbiol Mol Biol Rev (2012) 76:16–32. doi: 10.1128/MMBR.05015-11 PMC3294426 22390970

[10] Lu HQ Hu R. The Role of Immunity in the Pathogenesis and Development of Pre-Eclampsia. Scand J Immunol (2019) 90:e12756. doi: 10.1111/sji.12756 30739345

[11] Gong Q Zhu Y Pang N Ai H Gong X La X. Increased Levels of CCR7(lo)PD-1(hi) CXCR5(+) CD4(+) T Cells, and Associated Factors Bcl-6, CXCR5, IL-21 and IL-6 Contribute to Repeated Implantation Failure. Exp Ther Med (2017) 14:5931–41. doi: 10.3892/etm.2017.5334 PMC5740606 29285142

[12] Ghaebi M Abdolmohammadi-Vahid S Ahmadi M Eghbal-Fard S Dolati S Nouri M. T cell Subsets in Peripheral Blood of Women with Recurrent Implantation Failure. J Reprod Immunol (2019) 131:21–9. doi: 10.1016/j.jri.2018.11.002 30471511

[13] Yang KM Ntrivalas E Cho HJ Kim NY Beaman K Gilman-Sachs A. Women With Multiple Implantation Failures and Recurrent Pregnancy Losses Have Increased Peripheral Blood T Cell Activation. Am J Reprod Immunol (2010) 63:370–8. doi: 10.1111/j.1600-0897.2010.00811.x 20236264

[14] Zeng W Liu Z Liu X Zhang S Khanniche A Zheng Y. Distinct Transcriptional and Alternative Splicing Signatures of Decidual CD4(+) T Cells in Early Human Pregnancy. Front Immunol (2017) 8:682. doi: 10.3389/fimmu.2017.00682 28659920 PMC5466981

[15] Yuan J Li J Huang SY Sun X. Characterization of the Subsets of Human NKT-Like Cells and the Expression of Th1/Th2 Cytokines in Patients With Unexplained Recurrent Spontaneous Abortion. J Reprod Immunol (2015) 110:81–8. doi: 10.1016/j.jri.2015.05.001 26057526

[16] Puissant F Van Rysselberge M Barlow P Deweze J Leroy F. Embryo Scoring as a Prognostic Tool in IVF Treatment. Hum Reprod (1987) 2:705–8. doi: 10.1093/oxfordjournals.humrep.a136618 3437050

[17] Gardner DK Lane M Stevens J Schlenker T Schoolcraft WB. Blastocyst Score Affects Implantation and Pregnancy Outcome: Towards a Single Blastocyst Transfer. Fertil Steril (2000) 73:1155–8. doi: 10.1016/s0015-0282(00)00518-5 10856474

[18] Cha J Sun X Dey SK. Mechanisms of Implantation: Strategies for Successful Pregnancy. Nat Med (2012) 18:1754–67. doi: 10.1038/nm.3012 PMC6322836 23223073

[19] Tan Q Shi S Liang J Zhang X Cao D Wang Z. MicroRNAs in Small Extracellular Vesicles Indicate Successful Embryo Implantation during Early Pregnancy. Cells (2020) 9. doi: 10.3390/cells9030645 PMC7140406 32155950

[20] Wang Y Ma CH Qiao J. [Differential Expression of microRNA in Eutopic Endometrium Tissue During Implantation Window for Patients With Endometriosis Related Infertility]. Zhonghua Fu Chan Ke Za Zhi (2016) 51:436–41. doi: 10.3760/cma.j.issn.0529-567X.2016.06.007 27356479

[21] Gnainsky Y Granot I Aldo P Barash A Or Y Mor G. Biopsy-Induced Inflammatory Conditions Improve Endometrial Receptivity: The Mechanism of Action. Reproduction (2015) 149:75–85. doi: 10.1530/REP-14-0395 25349438

[22] Ledee N Petitbarat M Chevrier L Vitoux D Vezmar K Rahmati M. The Uterine Immune Profile May Help Women With Repeated Unexplained Embryo Implantation Failure After In Vitro Fertilization. Am J Reprod Immunol (2016) 75:388–401. doi: 10.1111/aji.12483 PMC4849202 26777262

[23] Yang F Zheng Q Jin L. Dynamic Function and Composition Changes of Immune Cells During Normal and Pathological Pregnancy at the Maternal-Fetal Interface. Front Immunol (2019) 10:2317. doi: 10.3389/fimmu.2019.02317 31681264 PMC6813251

[24] Sheikhansari G Pourmoghadam Z Danaii S Mehdizadeh A Yousefi M. Etiology and Management of Recurrent Implantation Failure: A Focus on Intra-Uterine PBMC-Therapy for RIF. J Reprod Immunol (2020) 139:103121. doi: 10.1016/j.jri.2020.103121 32240947

[25] Wang W Sung N Gilman-Sachs A Kwak-Kim J. T Helper (Th) Cell Profiles in Pregnancy and Recurrent Pregnancy Losses: Th1/Th2/Th9/Th17/Th22/Tfh Cells. Front Immunol (2020) 11:2025. doi: 10.3389/fimmu.2020.02025 32973809 PMC7461801

[26] Raghupathy R Kalinka J. Cytokine Imbalance in Pregnancy Complications and Its Modulation. Front Biosci (2008) 13:985–94. doi: 10.2741/2737 17981605

[27] Todt JC Yang Y Lei J Lauria MR Sorokin Y Cotton DB. Effects of Tumor Necrosis Factor-Alpha on Human Trophoblast Cell Adhesion and Motility. Am J Reprod Immunol (1996) 36:65–71. doi: 10.1111/j.1600-0897.1996.tb00141.x 8862248

[28] Haider S Knofler M. Human tumour necrosis factor: physiological and pathological roles in placenta and endometrium. Placenta (2009) 30:111–23. doi: 10.1016/j.placenta.2008.10.012 PMC2974215 19027157

[29] Seki H Matuoka K Inooku H Takeda S. TNF-Alpha From Monocyte of Patients With Pre-Eclampsia-Induced Apoptosis in Human Trophoblast Cell Line. J Obstet Gynaecol Res (2007) 33:408–16. doi: 10.1111/j.1447-0756.2007.00551.x 17688605

[30] Zhang Y Wang Y Li MQ Duan J Fan DX Jin LP. IL-25 Promotes Th2 Bias by Upregulating IL-4 and IL-10 Expression of Decidual gammadeltaT Cells in Early Pregnancy. Exp Ther Med (2018) 15:1855–62. doi: 10.3892/etm.2017.5638 PMC5776656 29434775

[31] Prins JR Gomez-Lopez N Robertson SA. Interleukin-6 in Pregnancy and Gestational Disorders. J Reprod Immunol (2012) 95:1–14. doi: 10.1016/j.jri.2012.05.004 22819759

[32] Hercor M Anciaux M Denanglaire S Debuisson D Leo O Andris F. Antigen-Presenting Cell-Derived IL-6 Restricts the Expression of GATA3 and IL-4 by Follicular Helper T Cells. J Leukoc Biol (2017) 101:5–14. doi: 10.1189/jlb.1HI1115-511R PMC5166434 27474166

[33] Sheikhansari G Soltani-Zangbar MS Pourmoghadam Z Kamrani A Azizi R Aghebati-Maleki L. Oxidative Stress, Inflammatory Settings, and microRNA Regulation in the Recurrent Implantation Failure Patients With Metabolic Syndrome. Am J Reprod Immunol (2019) 82:e13170. doi: 10.1111/aji.13170 31310689

[34] Amjadi F Zandieh Z Mehdizadeh M Aghajanpour S Raoufi E Aghamajidi A. The Uterine Immunological Changes May Be Responsible for Repeated Implantation Failure. J Reprod Immunol (2020) 138:103080. doi: 10.1016/j.jri.2020.103080 32120158

[35] Monin L Gaffen SL. Interleukin 17 Family Cytokines: Signaling Mechanisms, Biological Activities, and Therapeutic Implications. Cold Spring Harb Perspect Biol (2018) 10. doi: 10.1101/cshperspect.a028522 PMC5732092 28620097

[36] Beringer A Miossec P. Systemic Effects of IL-17 in Inflammatory Arthritis. Nat Rev Rheumatol (2019) 15:491–501. doi: 10.1038/s41584-019-0243-5 31227819

[37] McGeachy MJ Cua DJ Gaffen SL. The IL-17 Family of Cytokines in Health and Disease. Immunity (2019) 50:892–906. doi: 10.1016/j.immuni.2019.03.021 PMC6474359 30995505

[38] Kamath MS Chittawar PB Kirubakaran R Mascarenhas M. Use of Granulocyte-Colony Stimulating Factor in Assisted Reproductive Technology: A Systematic Review and Meta-Analysis. Eur J Obstet Gynecol Reprod Biol (2017) 214:16–24. doi: 10.1016/j.ejogrb.2017.04.022 28458165

[39] Wurfel W. Treatment With Granulocyte Colony-Stimulating Factor in Patients With Repetitive Implantation Failures And/or Recurrent Spontaneous Abortions. J Reprod Immunol (2015) 108:123–35. doi: 10.1016/j.jri.2015.01.010 25740726

[40] Asayama K Kobayashi T D'Alessandro-Gabazza CN Toda M Yasuma T Fujimoto H. Protein S Protects Against Allergic Bronchial Asthma by Modulating Th1/Th2 Balance. Allergy (2020) 75:2267–78. doi: 10.1111/all.14261 32145080

[41] Arif S Gomez-Tourino I Kamra Y Pujol-Autonell I Hanton E Tree T. GAD-Alum Immunotherapy in Type 1 Diabetes Expands Bifunctional Th1/Th2 Autoreactive CD4 T Cells. Diabetologia (2020) 63:1186–98. doi: 10.1007/s00125-020-05130-7 PMC7228993 32248243

[42] Lin H Mosmann TR Guilbert L Tuntipopipat S Wegmann TG. Synthesis of T Helper 2-Type Cytokines at the Maternal-Fetal Interface. J Immunol (1993) 151:4562–73. 8409418

[43] Saito S Nakashima A Shima T Ito M. Th1/Th2/Th17 and Regulatory T-Cell Paradigm in Pregnancy. Am J Reprod Immunol (2010) 63:601–10. doi: 10.1111/j.1600-0897.2010.00852.x 20455873

[44] Kwak-Kim JY Chung-Bang HS Ng SC Ntrivalas EI Mangubat CP Beaman KD. Increased T Helper 1 Cytokine Responses by Circulating T Cells Are Present in Women With Recurrent Pregnancy Losses and in Infertile Women With Multiple Implantation Failures After IVF. Hum Reprod (2003) 18:767–73. doi: 10.1093/humrep/deg156 12660269

[45] Liang PY Diao LH Huang CY Lian RC Chen X Li GG. The Pro-Inflammatory and Anti-Inflammatory Cytokine Profile in Peripheral Blood of Women With Recurrent Implantation Failure. Reprod Biomed Online (2015) 31:823–6. doi: 10.1016/j.rbmo.2015.08.009 26371706

