# Supplementary material for: Alterations of Cytokine Profiles in Patients With Recurrent Implantation Failure
Source: Front Endocrinol (Lausanne). 2022 Jul 11;13:949123. doi: 10.3389/fendo.2022.949123 (PMC9309554; doi:10.3389/fendo.2022.949123)
Supplement: Supplementary file 1 [file DataSheet_1.pdf]

Supplementary Material

Supplementary Figures

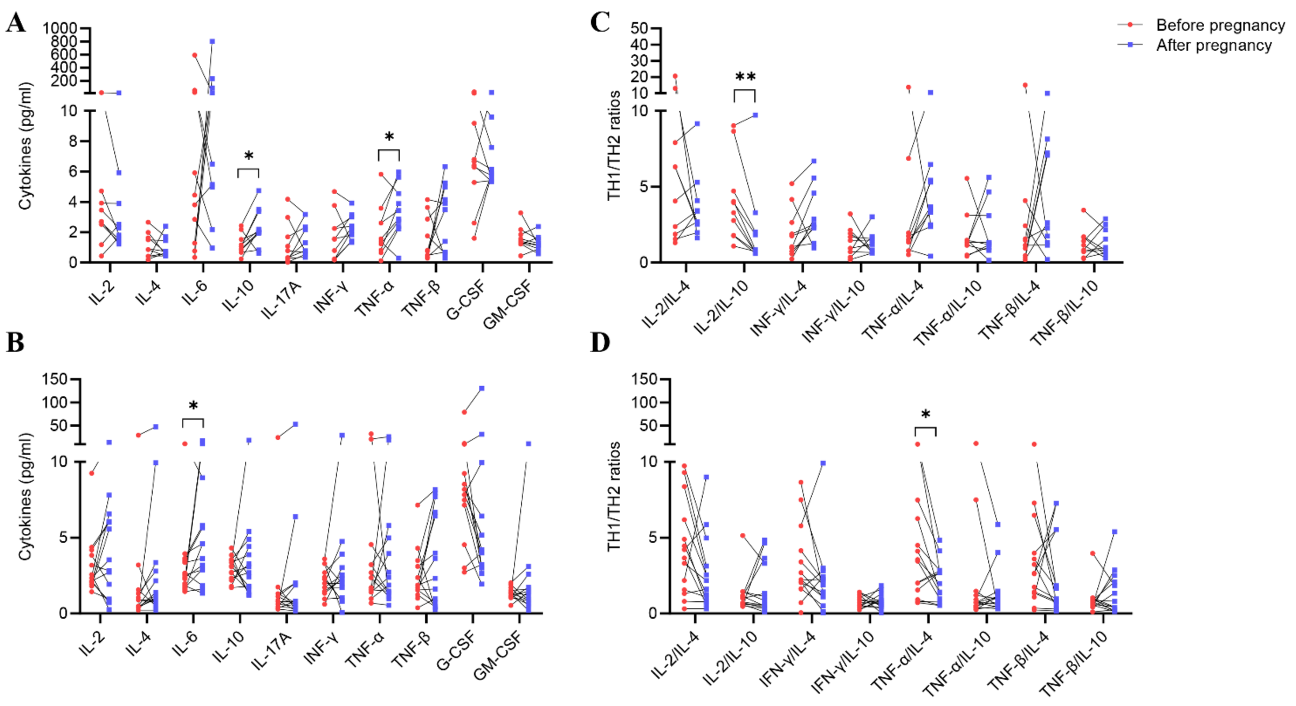

Supplementary Figure 1. Changes in serum cytokine profiles and Th1/Th2 ratios in the RIF and control groups before and after pregnancy. (A) Altered serum cytokines in the RIF group before and after pregnancy. (B) Altered serum cytokines in the control group before and after pregnancy. (C) Altered Th1/Th2 ratios of the RIF group before and after pregnancy. (D) Altered Th1/Th2 ratios of the control group before and after pregnancy. **P* < 0.05; ** *P* < 0.01; ****P* < 0.001; *****P* < 0.0001.
